# Assessment tools for disease risk perception in chronic patients: theoretical frameworks, psychometric properties, and clinical applications

**DOI:** 10.3389/fpubh.2026.1821777

**Published:** 2026-05-08

**Authors:** Fei Yang, Jiaojian Lv, Qiaoling Ye, Lihong Jin, Yuanliang Gu, Jinxiang Wu

**Affiliations:** 1 Medicine School of Lishui University, Lishui, Zhejiang, China; 2 Department of Hepatology and Infectious Diseases, The First Affiliated Hospital of Lishui University, Lishui People’s Hospital, Lishui, Zhejiang, China; 3 Department of Public Health, The First Affiliated Hospital of Lishui University, Lishui People’s Hospital, Lishui, Zhejiang, China

**Keywords:** assessment tools, chronic disease, disease risk perception, psychometric properties, theoretical framework

## Abstract

Disease risk perception is a subjective psychological construct that can predict health-promoting behaviors and support personalized chronic disease management. This narrative review summarizes existing risk perception assessment tools, analyzing their theoretical foundations, structures and psychometric properties. We trace the evolution of these tools from broad, generic scales to disease-specific evaluations for conditions such as cardiovascular disease, stroke, cancer and diabetes. The review also examines the supporting theoretical frameworks, such as the Health Belief Model, Risk Perception Theory, Optimism Bias Theory and the Dual-Process Model. There has been a shift from unidimensional to multidimensional psychological constructs, integrating both rationality and emotion. The evolution from generic to disease-specific assessments enables more targeted insights. However, current instruments face challenges such as insufficient cross-cultural validation, limited integration of emotional and cognitive factors, and inconsistent predictive validity for health behaviors. It is recommended that future research efforts concentrate on the development of comprehensive tools. It is imperative that these tools take into consideration sociocultural contexts and dual-processing mechanisms. The implementation of such tools has the potential to enhance the precision and predictive capacity of disease risk perception assessments, thereby facilitating the optimization of patient-centered interventions.

## Introduction

Risk perception is defined as the empirical understanding an individual forms following a subjective—and often non-rational—analysis of environmental risk information, playing a crucial role in the initiation and maintenance of health-promoting behaviors (1). Far from being a mere mirror reflection of objective statistical probability, it is a complex psychological construct that integrates cognitive interpretation, affective response, personal experience, social propagation, and cultural context (2). Substantial empirical evidence confirms that accurate and moderate risk perception acts as a pivotal internal lever (3–5); conversely, significant deviations in risk perception—whether underestimations driven by optimistic bias or information deficits leading to behavioral inertia, or over estimations caused by excessive fear resulting in decision paralysis and diminished quality of life—exert profound negative impacts on both individual health outcomes and healthcare system efficiency (6, 7).

Against this backdrop, the scientific and effective assessment of disease risk perception has become increasingly critical. Under the modern paradigm of “patient-centered” care, precise risk perception assessment serves as the cornerstone for personalized health management, optimized doctor-patient communication, and enhanced intervention efficacy. It functions dualistically: first, as a “diagnostic tool” for clinicians to gain insight into patients’ internal mental models and identify cognitive misconceptions or affective needs (8); and second, as an indispensable “standardized metric” for evaluating the effectiveness of health education, measuring adherence to behavioral interventions, and conducting high-quality clinical research (5). Driven by these compelling clinical applications, the development and validation of assessment tools with robust reliability, validity, and responsiveness have become a vibrant and rapidly evolving research focus.

The evolution of risk perception assessment tools demonstrates a clear trajectory from broad exploration to deep focus. In the early stages of research, the development of generic scales was prioritized. Examples of such scales include the Chronic Patients’ Risk Perception Questionnaire (CPRP) (9) from China and the Tripartite Model of Risk Perception (TRIRISK) (10) from the United States. The objective of the design of both of these tools was to facilitate the assessment of subjective risk perceptions across a variety of health conditions, with no focus on specific diseases. The CPRP, developed to evaluate how patients with chronic diseases perceive various medical risks, covers dimensions such as economic, physical, and psychosocial risks. Similarly, the TRIRISK instrument, developed by Ferrer et al. (10), aimed to explore the universal psychological dimensions of risk perception. This encompasses deliberative, affective and experiential components across various health conditions. Whilst these generic tools furnished a rudimentary framework for cross-disease comparison, clinical practice has demonstrated that the considerable heterogeneity in etiology, progression, symptom experience, therapeutic modalities and social symbolism across different diseases necessitates unique, disease-specific risk perception structures. This realization has catalyzed the proliferation of disease-specific tools, refining the research scope from chronic diseases in general (11) to specific pathologies (12), target populations (13), and even distinct treatment phases (14, 15), thereby achieving greater precision and contextual relevance.

Notably, over the past several decades, scholars from a variety of regions have made significant contributions to this field. Researchers in Myanmar developed the Non-communicable Diseases Perceived Risk (NCD-PR5-21) (16), which is tailored to local non-communicable disease risks. Investigators in the Netherlands (17), China (18, 19), Belgium and England (20), Jordan (21), and Egypt (22) have culturally adapted and validated the ABCD Risk Questionnaire (23), while Chinese scholars have developed and validated a series of localized scales for conditions such as stroke, cancer and diabetes (12, 24, 25). These scales address the region’s specific cultural context, disease spectrum and medical practices. These global efforts not only enrich the repository of assessment tools, but also highlight a trend towards diversification and localization in risk perception research.

However, despite the abundance of emerging assessment tools, there remains a paucity of systematic reviews that organize, compare, and integrate these diverse instruments. The variations in theoretical foundations, dimensional compositions, target populations, scoring methods, and psychometric properties among these tools pose significant challenges for researchers selecting appropriate instruments and for clinicians understanding their application scenarios. Therefore, this review aims to provide a comprehensive and systematic inventory and integrated analysis of existing disease risk perception assessment tools. We first delineate the developmental lineage from generic to specific tools, then detail their applications and characteristics in major fields such as cardiovascular disease, stroke, oncology, and maternal health. Finally, based on a comparative analysis, we discuss current limitations and future frontiers, providing a reference for scholars and practitioners to enhance the precision of patient assessment and the effectiveness of health interventions.

### Concepts and origins of risk perception

The theoretical study of risk perception originated in the field of consumer behavior. In 1960, Bauer from Harvard University first introduced the concept of “perceived risk,” defining it as the cognition of uncertainty regarding consequences in consumer decision-making (26). Subsequently, Cox and Cunningham further refined the internal structure of this concept (27, 28), proposing the two-factor model and the multidimensional model, respectively. The former emphasizes that risk perception is jointly constituted by the uncertainty of an event’s occurrence and the severity of its consequences. In contrast, the latter posits that, in addition to these factors, dimensions such as individual psychological states and coping processes must be incorporated. In the Chinese context, Dai et al. (29), based on the integration of international theories and systematic expert argumentation, synthesized risk perception as a subjective judgment made by individuals regarding the probability, severity, and controllability of objective risks, grounded in sensory emotions and life experiences.

Since the 1990s, research on risk perception has progressively extended into the health domain. In 1990, Blalock et al. pioneered this transition by exploring the impact of risk perception on screening behaviors among colorectal cancer patients (30). Later, in 1996, Rich et al. further analyzed the relationship between HIV risk perception, knowledge levels, and risk behaviors among college students (31). With the deepening of research, the field has currently evolved along two primary trajectories: one focuses on the mechanistic associations between risk perception and high-risk behaviors such as smoking, alcohol abuse, and unsafe sexual practices (32–34); the other progressively explores the measurement, assessment, and intervention of risk perception specifically regarding chronic diseases (35–37).

Disease risk perception refers to an individual’s judgment of the magnitude of their risk for developing a disease, based on subjective cognition and understanding (24). Assessing disease risk perception can stimulate disease prevention awareness, increase screening rates, and promote the active adoption of preventive behaviors (38, 39). Theoretically, individuals may overestimate the risk of disease occurrence, leading to unnecessary and detrimental chronic anxiety; conversely, they may underestimate the risk, thereby diminishing their motivation to adopt coping behaviors and preventive measures (40). Therefore, the objective and accurate assessment of patients’ disease risk perception is of paramount importance for the implementation of targeted health education and guidance.

**Figure 1:**
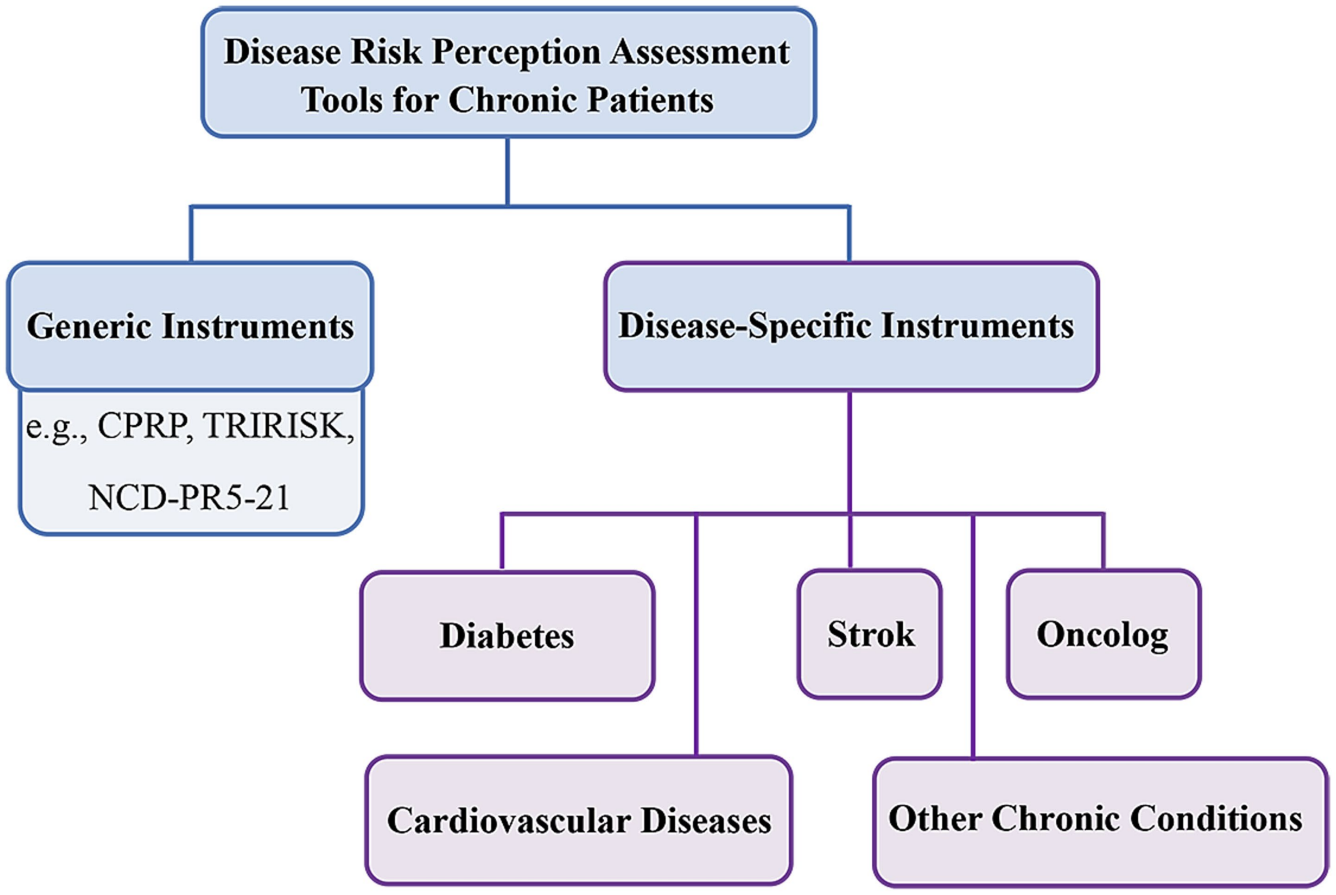
Organizational framework of reviewed assessment tools. CPRP: Chronic Patients’ Risk Perception Questionnaire; TRIRISK: The Tripartite Model of Risk Perception; NCD-PR5-21: Non-communicable Diseases Perceived Risk. Classification framework of existing disease risk perception assessment tools reviewed in this study. The diagram illustrates the categorization into generic versus disease-specific instruments, with further stratification of specific tools based on chronic disease typology.

Figure 1 illustrates the organizational framework of the assessment tools categorized in this study, to visually guide readers through the scope of this review. This figure provides a structured overview of both generic and disease-specific scales, bridging the theoretical foundations discussed above with the subsequent detailed review of existing instruments.

## Methods

### Aims

The aim of this review was to identify, critically appraise and summarize instruments that measure disease risk perception among patients with chronic diseases in healthcare settings.

### Search strategy

A comprehensive search was conducted using both Chinese and English databases, with consideration given to the significant population size and linguistic universality of the two languages. The Wan fang, China National Knowledge Infrastructure (CNKI), China Science and Technology Journal (VIP), PubMed, Cochrane Library, Web of Science, Embase and Cumulative Index to Nursing and Allied Health Literature (CINAHL) databases were searched from inception until October 30, 2025, using the following keywords:“risk perception,”“risk factors,”“perceived worry,”“Surveys and Questionnaires,”“measure,”“tool,” etc.

### Eligibility criteria

Studies were eligible for inclusion if they met the following criteria: (a) original studies and adaptations that developed or validated a chronic disease risk perception instrument in patients with chronic diseases; (b) studies that described at least one of the measurement properties of chronic disease risk perception instruments; and (c) articles that were published in Chinese or English.

### Study selection and screening

The records were imported into a reference management tool (Zotero, version 7). The screening process was conducted independently by two authors (FY and Lj). Initially, duplicate studies were removed, after which the remaining studies were assessed based on their titles and abstracts to determine their eligibility. After applying the inclusion criteria, a full-text review was conducted, and the reference lists of all eligible studies were examined to identify any potentially relevant additional studies. Where there was disagreement about which studies to select, a deliberative process involving three authors (FY, Lj and Wj) was initiated to reach a consensus.

### Data extraction

Relevant information from included studies was extracted independently by 2 reviewers (FY and Lj) using a data charting form. Charted information was then cross-checked to ensure all necessary details were accurate. The following data were charted: article features (author, year, country), population, context (target population and theoretical basis) and features of the assessment tools (name, details of development or modification, content, structure, number of items, validity and reliability of tool).

## Results

### Study selection

**Figure 2:**
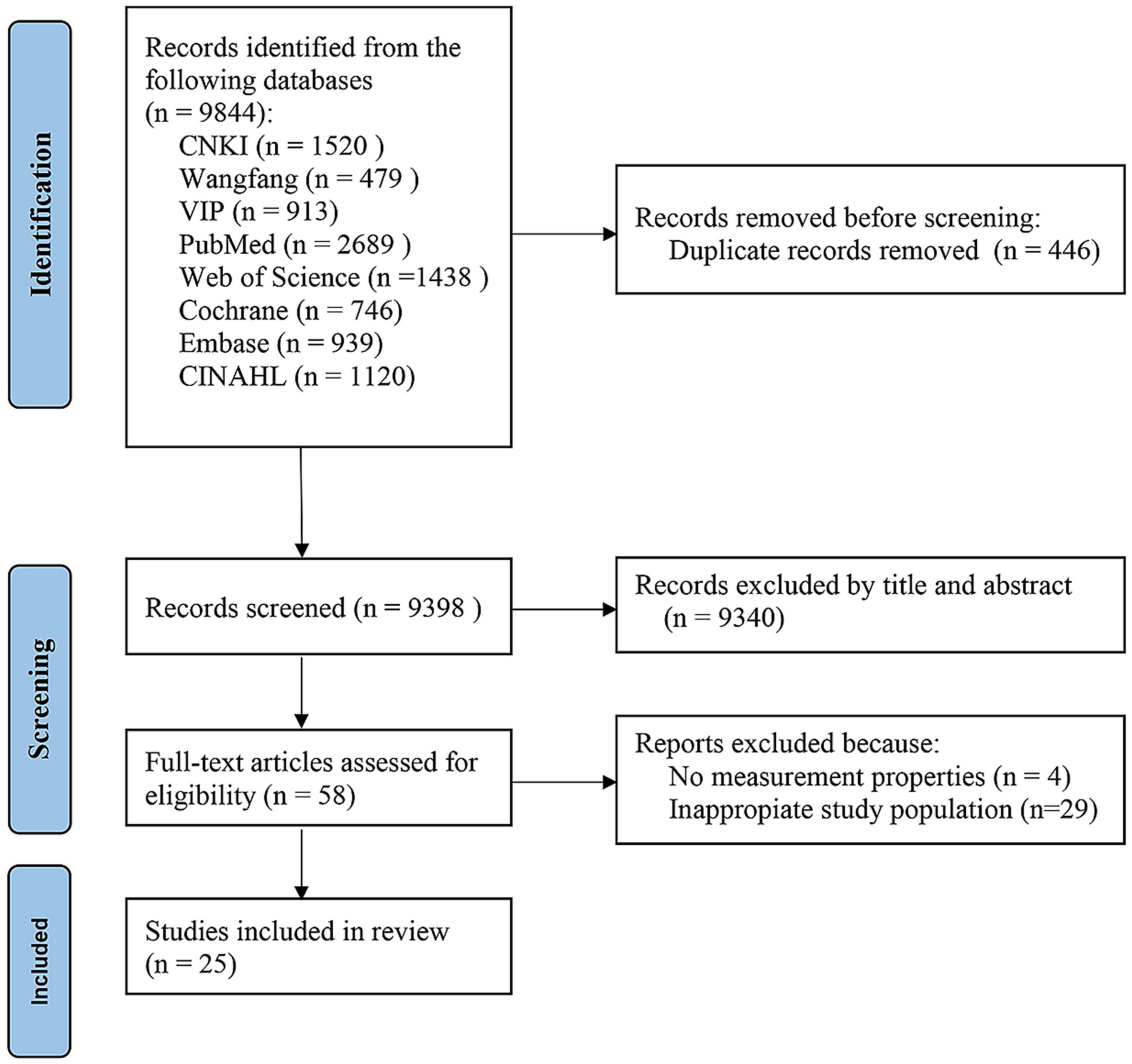
PRISMA flow diagram of the literature search and selection process|.

Figure 2 shows the PRISMA 2020 guideline flowchart. The preliminary database search yielded 9,844 references. After removing duplicates, 9,398 records were retained across all databases, and a total of 9,398 titles and abstracts were reviewed. After applying the pre-determined eligibility criteria, 9,340 studies were excluded and the full-text articles of 58 studies were assessed for eligibility. The final analysis encompassed a total of 25 studies, which were subjected to rigorous evaluation using the COSMIN Risk of Bias tool to ascertain the quality of these studies.

### Summary of assessment tools

#### Generic assessment tools for disease risk perception

##### Chronic patients’ risk perception questionnaire (CPRP)

The Chronic Patients’ Risk Perception Questionnaire (CPRP) was developed by the Fang (9) in 2015 to evaluate the subjective risk perception of patients with chronic diseases regarding various hazards encountered during medical processes. The instrument comprises 12 items distributed across three dimensions: economic risk, physical diagnosis and treatment risk, and psychosocial risk. Items are rated on a 5-point Likert scale, yielding a theoretical total score range of 12 to 60, where higher scores indicate elevated levels of risk perception. Psychometric testing demonstrated robust reliability, with a total Cronbach’s *α* coefficient of 0.884 and dimensional coefficients ranging from 0.826 to 0.870; the test–retest reliability was 0.9824. Owing to its rigorous development process and demonstrated favorable validity and stability, the CPRP has been widely applied in studies assessing risk perception among patients with diabetes (41), breast cancer (42), stroke (12), and those undergoing hemodialysis (43).

##### The tripartite model of risk perception (TRIRISK)

The Tripartite Model of Risk Perception (TRIRISK) was developed by Ferrer et al. (10) in 2016 to evaluate the public’s subjective perception of the risk of developing chronic diseases. This instrument comprises 18 items distributed across three dimensions: deliberative, affective, and experiential perception. With the exception of Item 2, which utilizes a numerical scale, all items are scored on a 7-point Likert scale, where higher scores indicate elevated levels of risk perception. Psychometric testing revealed Cronbach’s *α* coefficients ranging from 0.88 to 0.96 for the individual dimensions and 0.93 for the total scale, demonstrating robust reliability and good model fit. The scale was initially validated across three populations—cancer, heart disease, and diabetes—where results indicated that while it possessed good discriminative validity for heart disease risk perception, its discrimination was less optimal for cancer and diabetes populations. In 2018, Ferrer et al. (44) conducted further research to elucidate the relationship between these risk perception dimensions and individual protection motivation. Empirical analysis demonstrated intercorrelations among the three dimensions, identifying affective risk perception as the strongest predictor of protection motivation. Furthermore, the predictive validity of these dimensions was found to be moderated by individual differences and environmental factors: deliberative risk perception was more predictive when the individual’s need for cognition was high; affective risk perception was more predictive when the need for affect was high; and experiential risk perception was more predictive when coping confidence was high. This study confirmed the correlation between multidimensional risk perception and protection motivation, providing an evidence base for tailoring interventions to individual differences. In 2025, Zhou et al. (45) completed the Chinese localization of the scale; validation among family members of patients with digestive tract cancer yielded Cronbach’s *α* coefficients of 0.906–0.976 for the dimensions and 0.953 for the total scale, indicating that the Chinese version possesses good reliability and validity for assessing comprehensive disease risk perception in the Chinese population. However, a study by Riedinger et al. (46) applied the scale to a UK cancer population and found that the deliberative dimension further decomposed into “numerical” and “reflective” sub-dimensions, while cross-loading occurred between affective and experiential items, suggesting that the scale’s cultural adaptability requires further verification.

##### Non-communicable diseases perceived risk (NCD-PR5-21)

The Non-communicable Diseases Perceived Risk (NCD-PR5-21) assessment tool was developed by Mya et al. (16) in 2021 to evaluate subjective risk perceptions regarding four major non-communicable diseases (NCDs) among the adult population in Myanmar. Constructed upon the Health Belief Model (47), the instrument encompasses five core dimensions: perceived susceptibility, perceived benefits, perceived barriers, self-efficacy, and intention for behavioral change. The questionnaire comprises 21 items rated on a 4-point Likert scale, yielding a total score range of 21 to 84, where higher scores indicate elevated levels of risk perception. All items were designed to target common risk behaviors associated with four specific categories of diseases: cardiovascular disease, diabetes, cancer, and chronic respiratory diseases. The Cronbach’s *α* coefficients for the dimensions ranged from 0.683 to 0.854; with the exception of the perceived barriers dimension, which was marginally lower, all dimensions met standard psychometric requirements. Developed based on multidimensional psychological constructs, this scale effectively discriminates between facilitating and inhibiting factors within the behavioral change process, thereby providing an evidence base for the formulation and optimization of intervention protocols. However, as the scale was developed recently in 2021, its application is currently limited to a specific cultural context, and its cross-cultural universality remains unverified. Therefore, future research involving cross-cultural adaptation and validation is urgently needed to establish its broader applicability.

##### Risk perception of chronic disease (RPCD)

The Risk Perception of Chronic Disease (RPCD) questionnaire was developed by the American scholar Hamilton et al. (11) in 2012 to assess subjective perceptions of chronic disease risk among young and middle-aged women. The instrument comprises four dimensions: perceived personal risk, perceived risk to others, estimation of disease prevalence, and estimation of disease mortality. Tailored to three specific chronic conditions—cardiovascular disease, breast cancer, and lung cancer—each dimension specifically measures the individual’s level of risk perception regarding these diseases. For scoring, the dimensions of “perceived personal risk” and “perceived risk to others” utilize a 5-point Likert scale, with values ranging from 0 (“impossible”) to 4 (“very high probability”); conversely, “prevalence estimation” and “mortality estimation” employ an absolute numerical scale ranging from 0 to 100, where higher scores indicate a greater perceived likelihood of developing the disease. The Cronbach’s *α* coefficients for the dimensions ranged from 0.62 to 0.825. While this scale effectively elucidates the multidimensional psychological nature of risk perception, it is notably focused on the female population; consequently, it presents limitations regarding the gender specificity of item content, and given that its development process was not elaborated in detail, the credibility of its reliability warrants further verification.

##### Risk perception scale of disease aggravation for older patients

The Risk Perception Scale of Disease Aggravation for Older Patients was developed by Wang et al. (48) in 2023 within the Chinese socio-cultural context to assess the subjective perception of disease aggravation risk among older adult patients with chronic conditions. Constructed upon the theoretical foundations of Cognitive-Experiential Self-Theory (18), Affect Heuristic Theory (19), and Risk Perception Theory (49, 50), the instrument comprises 40 items distributed across four dimensions: affective experience, possible causes, severe consequences, and behavioral control. All items are rated using a 5-point Likert scale, yielding a total score range of 40–200, where higher scores indicate a higher overall level of perceived risk regarding disease aggravation. Reliability analysis demonstrated Cronbach’s *α* coefficients ranging from 0.920 to 0.973 for the individual dimensions and 0.973 for the total scale. As the first generic instrument in China specifically targeting the perception of disease aggravation risk in older adult chronic disease patients1, its development process was scientifically rigorous; however, its psychometric properties require further refinement through validation in broader populations and longitudinal studies. A summary of the key features and psychometric properties of the reviewed generic assessment tools is presented in Table 1.

**Table 1:** Characteristics and psychometric properties of generic disease risk perception assessment tools.

Instrument name	Author (Year) (Ref)	Target population	Theoretical basis	Key dimensions (Items)	Reliability (Cronbach’s *α*)
CPRP (Chronic patients’ risk perception questionnaire)	Fang (2015) (9)	Chronic disease patients	Literature Review / Expert Consultation	3 Dimensions (12 items): Economic risk, Physical diagnosis/treatment risk, Psychosocial risk.	Total: 0.88Dims: 0.83–0.87
TRIRISK (The tripartite model of risk perception)	Ferrer et al. (2016)(10)	General public (Adults)	Risk Perception Theory	3 Dimensions (18 items): Deliberative, Affective, Experiential perception.	Total: 0.93Dims: 0.88–0.96
NCD-PR5-21 (Non-communicable diseases perceived risk)	Mya et al. (2021)(16)	Adults (Myanmar)	Health Belief Model (HBM)	5 Dimensions (21 items): Susceptibility, Benefits, Barriers, Self-efficacy, Intention for change.	Dims: 0.68–0.85
RPCD (Risk perception of chronic disease)	Hamilton et al. (2012)(11)	Young and Middle-aged women	N/A	4 Dimensions: Personal risk, Risk to others, Prevalence estimation, Mortality estimation.	Dims: 0.62–0.82
Risk Perception Scale of Disease Aggravation	Wang et al. (2023)(48)	Older adults with chronic diseases	CEST, Affect Heuristic, Risk Perception Theory	4 Dimensions (40 items): Affective experience, Possible causes, Severe consequences, Behavioral control.	Total: 0.97Dims: 0.92–0.97

CEST = Cognitive-Experiential Self-Theory. Dimensions refer to the structural components of the scale.

### Disease-specific risk perception assessment tools

#### Assessment tools for cardiovascular disease risk perception

##### Perceived risk of coronary heart disease (PRCHD)

The Perceived Risk of Coronary Heart Disease (PRCHD) instrument was developed by Becker and Levine (51) in 1987. Constructed based on the Health Belief Model (47), this questionnaire is designed to assess the level of risk perception and lifestyle-associated risks among the unaffected siblings of patients with premature coronary heart disease (CHD). The scale comprises four items covering absolute risk, relative risk, and personal worry. Specifically, absolute risk includes two items assessing the probability of developing CHD within the next 10 years and over a lifetime; relative risk is measured by one item comparing the individual’s probability to that of peers of the same age and gender; and an additional item measures the frequency of worry, resulting in a four-dimensional structure. The relative risk item utilizes a 5-point Likert scale (scored 1–5), ranging from “much lower” to “much higher.” The remaining items employ a 6-point Likert scale (scored 0–5), with anchors ranging from “not at all worried” or “very low possibility” to “very worried” or “very high possibility”. The total score ranges from 0 to 20, with higher scores indicating a heightened level of perceived risk. The Cronbach’s *α* coefficient for the scale was reported as 0.803. Validation studies conducted in Korean populations (52, 53) yielded Cronbach’s α coefficients between 0.78 and 0.86, with results suggesting a generally low level of risk perception in this demographic. However, a limitation of this instrument is its predominant focus on assessing disease susceptibility1, with insufficient evaluation regarding the perception of disease severity and intentions for behavioral change.

##### • The Perception of Risk of Heart Disease Scale (PRHDS)

The Perception of Risk of Heart Disease Scale (PRHDS) was co-developed by the Jordanian scholar Ammouri and the American scholar Neuberger in 2008 (54) to assess an individual’s subjective perception of heart disease risk. Constructed upon the psychometric paradigm proposed by Slovic (55), the scale comprises three dimensions: dread risk, general risk, and unknown risk. Each dimension assesses distinct facets of an individual’s heart disease risk perception through corresponding items. All items are rated on a 4-point Likert scale, ranging from 1 (“strongly disagree”) to 4 (“strongly agree”), where higher aggregate scores indicate a heightened perception of personal risk for developing heart disease. The Cronbach’s *α* coefficients for the dimensions ranged from 0.68 to 0.80, with a total scale coefficient of 0.80. The scale has been subsequently validated in the United States (56), Jordan (57), Italy (58), and Nigeria (59). In 2025, the Chinese scholar Luo (60) conducted a cultural adaptation and psychometric evaluation to provide a valid instrument for assessing subjective risk perception among the Chinese population free of heart disease. The original scale comprises 20 items; however, during the localization process, items 3 and 6 were removed following item analysis due to low item-total correlation, resulting in a final 18-item Chinese version. Scoring utilizes a 4-point Likert scale, with items 8 through 18 reverse-scored; the total score is the summation of all item scores, with higher values signifying elevated levels of heart disease risk perception. The Cronbach’s *α* coefficients for the dimensions ranged from 0.830 to 0.862, and the total scale coefficient was 0.847. The Chinese version of the PRHDS adhered to standardized cross-cultural adaptation procedures, and with its reliability and construct validity preliminarily verified, it offers a viable tool for assessing heart disease risk perception in the Chinese population.

##### Coronary risk individual perception (CRIP)

The Coronary Risk Individual Perception (CRIP) scale was developed by Barnhart et al. (61) in 2009 to evaluate the subjective perception of coronary heart disease (CHD) risk among urban minority adults. The instrument comprises 16 items distributed across four dimensions: worry about heart disease, self-efficacy, perceived susceptibility/vulnerability, and perceived health status. Items are rated on a 6-point Likert scale ranging from 1 (“strongly disagree”) to 6 (“strongly agree”). The total score ranges from 16 to 96, with higher scores indicating a heightened level of CHD risk perception. The scale demonstrated a total Cronbach’s *α* coefficient of 0.76. While the multidimensional structure of the CRIP facilitates a deeper understanding of the complex relationship between risk perception and health behaviors, its application and validation since its inception in 2009 have remained largely confined to the original urban minority population in the United States. To date, it has not undergone independent cross-cultural validation in other national or cultural settings. Consequently, future research should prioritize the adaptation and validation of the scale within diverse cultural contexts to examine its cross-cultural measurement equivalence.

##### • Attitude and beliefs about cardiovascular disease knowledge and risk questionnaire (ABCD risk questionnaire)

The Attitude and Beliefs about Cardiovascular Disease Knowledge and Risk Questionnaire (ABCD Risk Questionnaire) was developed by Woringer *et al*. (23) in 2017. Grounded in the Health Belief Model (47) and the Transtheoretical Model (62), this instrument was designed to assess the subjective level of cardiovascular disease (CVD) risk perception among high-risk populations within the National Health Service (NHS) in England. The questionnaire aims to evaluate patients’ accuracy of risk perception, mastery of related knowledge, and intentions for behavioral change following participation in the “NHS Health Check”. The final instrument comprises 26 items distributed across four dimensions: knowledge of CVD risk and prevention, perceived risk of heart attack/stroke, perceived benefits and intention to change behavior, and intention to eat healthily.

Scoring varies by dimension: the “knowledge of CVD risk and prevention” items are dichotomously scored (1 for correct, 0 for incorrect or “do not know”), while the remaining three dimensions utilize a 4-point Likert scale. Items 15, 21, and 26 are reverse-scored. The total score ranges from 0 to 80, with higher scores indicating a higher level of risk perception. Regarding reliability, the dimensions of “perceived risk” and “perceived benefits and intention to change” demonstrated robust internal consistency, with Cronbach’s *α* coefficients of 0.85 and 0.82, respectively; although the “intention to eat healthily” dimension yielded a lower Cronbach’s α of 0.56 due to its brevity (containing only three items), it was deemed acceptable for a short scale.

The ABCD scale underwent localization and psychometric validation in the Netherlands (17), where the structure was adjusted to four dimensions: CVD prevention knowledge, risk perception, perceived benefits and intention to change physical activity, and perceived benefits and intention to change diet. Reliability testing for the latter three dimensions (constituting the brief ABCD questionnaire) yielded a Cronbach’s *α* of 0.76, showing a high correlation with the original scale. To date, the scale has undergone cultural adaptation in the Netherlands (17), China (18, 19), Malaysia (63), Indonesia (64), Belgium and England (20), Jordan (21), and Egypt (22). While the scale effectively reflects the psychological constructs of CVD risk perception, it lacks specificity for populations exclusively at risk for heart disease.

#### Assessment tools for stroke risk perception

##### Stroke patient recurrence risk perception assessment scale

The Stroke Patient Recurrence Risk Perception Assessment Scale was developed by the Chinese scholar Lin *et al*. (65) in 2021 specifically to evaluate the subjective perception of disease recurrence risk among stroke patients. The scale is constructed upon the theoretical foundations of the Health Belief Model (47) and the Risk Perception Attitude Framework (66). The instrument comprises two sections with a total of 20 items. Section 1 consists of three items assessing the probability of recurrence: Item 1 requires patients to self-assess their recurrence risk level on a scale of 1 (“no risk”) to 5 (“high risk”); Items 2 and 3 evaluate the likelihood of recurrence within the next 1 and 10 years, respectively. Notably, if a patient selects “do not know” or “no risk” for Item 1, Items 2 and 3 are skipped and scored as 0; the total score for this section ranges from 1 to 15. Section 2 includes 17 items distributed across three dimensions: perceived severity of recurrence, perceived disease risk factors, and perceived behavioral risk factors. These items utilize a 3-point Likert scale ranging from 1 (“disagree”) to 3 (“agree”), yielding a score range of 17 to 51. The aggregate score for the entire scale ranges from 18 to 66, with higher scores indicating a stronger awareness of recurrence risk. Psychometric testing showed that Section 2 had a Cronbach’s *α* coefficient of 0.850, and the scale demonstrated good content validity and short-term stability. However, the split-half reliability for certain dimensions was low, and the 1-month test–retest reliability declined significantly, suggesting limitations in long-term stability which may be attributed to the situational changes patients experience when transitioning from hospital to home settings. Consequently, its stability and utility in long-term follow-up and intervention studies require further verification.

##### Recurrence risk perception scale for patients with ischemic stroke

The Recurrence Risk Perception Scale for Patients with Ischemic Stroke was developed by the Chinese scholar Han et al. (12) in 2022 to specifically assess the subjective perception of disease recurrence risk among ischemic stroke patients. The instrument is grounded in the theoretical frameworks of Risk Perception Theory (27, 28) and the Health Belief Model (47). It comprises 25 items distributed across four dimensions: warning symptoms, risk factors, health risks, and psychosocial risks. Items are rated on a 5-point Likert scale ranging from 1 (“strongly disagree”) to 5 (“strongly agree”), yielding a total score range of 5 to 125, where higher scores indicate a heightened level of recurrence risk perception. Psychometric analysis demonstrated Cronbach’s *α* coefficients ranging from 0.832 to 0.946 for the individual dimensions and 0.905 for the total scale. However, the complexity of certain item phrasings may compromise patient comprehension and response accuracy; consequently, its universality warrants further validation through multi-center studies.

##### Risk perception questionnaire for people at high risk of stroke

The Risk Perception Questionnaire for People at High Risk of Stroke was developed by the Chinese scholar Ren et al. (67) in 2023 to evaluate the subjective perception of stroke onset risk among high-risk populations. The questionnaire was constructed based on the Psychometric Paradigm (55) and the Health Belief Model (47). It encompasses 30 items across five dimensions: perceived susceptibility, perceived warning symptoms, perceived risk factors, perceived risk controllability, and perceived severity. All items are scored on a 5-point Likert scale ranging from 1 (“strongly disagree”) to 5 (“strongly agree”), resulting in a total score range of 30 to 150, where higher scores reflect elevated levels of risk perception. The Cronbach’s *α* coefficients for the dimensions ranged from 0.803 to 0.906, with an overall coefficient of 0.878. This instrument provides a specific assessment tool tailored for stroke risk perception among high-risk populations in China.

#### Assessment tools for cancer-related risk perception

##### Breast cancer recurrence risk perception assessment questionnaire

The Breast Cancer Recurrence Risk Perception Assessment Questionnaire was developed by Li et al. (68) in 2023 to evaluate the subjective perception of disease recurrence risk among breast cancer patients. Constructed upon the theoretical frameworks of the Health Belief Model (47) and the Risk Perception Attitude Framework (66), the instrument comprises 37 items distributed across six dimensions: perceived likelihood, perceived warning symptoms of recurrence, perceived disease risk factors, perceived behavioral risk factors, perceived psychological risk factors, and perceived severity. Items are rated using a 5-point Likert scale ranging from 1 (“never”) to 5 (“always”), yielding a total score between 37 and 185, where higher scores indicate a heightened level of recurrence risk perception. Cronbach’s *α* coefficients for the dimensions ranged from 0.82 to 0.90, with a total scale coefficient of 0.94. This questionnaire facilitates a systematic evaluation of multidimensional recurrence risk perception in breast cancer patients, providing an evidence base for risk communication and health behavior interventions.

##### Lymphedema risk perception questionnaire for female breast cancer patients

The Lymphedema Risk Perception Questionnaire for Female Breast Cancer Patients was developed by Ma *et al*. (69) in 2023 to assess the subjective perception of lymphedema risk among female breast cancer patients. Grounded in the Dual-Process Model framework (70), the questionnaire encompasses 22 items across three dimensions: cognitive judgment, emotional response, and perceived severity. Higher scores reflect elevated levels of risk perception. Reliability analysis demonstrated Cronbach’s *α* coefficients ranging from 0.870 to 0.893 for the dimensions, while the total scale coefficient was 0.745. By integrating both cognitive and affective processes, the scale covers the risk factors and severe consequences of lymphedema while emphasizing patients’ subjective feelings and emotional characteristics. The inclusion of psychological representation dimensions provides a scientific basis for evaluating the subjective perception of lymphedema risk among Chinese female breast cancer patients.

##### Visual analog scale (VAS)

The Visual Analogue Scale (VAS) was applied by researchers Hajian-Tilaki and Nikpour (71) in 2021 to assess breast cancer risk perception among Iranian women. As a single-item direct measurement tool, it quantifies an individual’s subjective judgment of their own breast cancer risk. The scale utilizes a 10-cm continuous line corresponding to a score range of 0 to 100, anchored by “no risk” and “absolute certainty” (or “high risk”). Participants mark the position on the line that reflects their perception, with higher scores indicating higher self-assessed risk. This method was utilized to obtain subjective ratings for both 5-year and lifetime risks. However, as a unidimensional instrument, the VAS presents significant limitations regarding the accuracy of risk perception and clinical applicability. Notably, its cross-cultural application may be susceptible to pessimistic bias, suggesting that it should be employed in conjunction with more structured risk assessment tools.

##### Skin cancer risk perception questionnaire (Morales-Sánchez et al.)

The Skin Cancer Risk Perception Questionnaire was developed by Morales-Sánchez *et al*. (72) in 2014 to assess an individual’s subjective perception regarding the risk of developing skin cancer. Constructed upon the theoretical framework of Risk Perception Theory (49, 50), the final instrument comprises 18 items organized into four distinct factors—affect, behavior, severity, and susceptibility—along with a single probability indicator. Items are rated using a 7-point Likert scale, yielding a total score range of 18 to 126, where higher scores denote an elevated level of risk perception. Psychometric analysis revealed Cronbach’s *α* coefficients ranging from 0.647 to 0.884 for the individual dimensions, with a total scale coefficient of 0.824. However, given that the validation study was primarily conducted among first-time patients at a specialized dermatology hospital in Mexico, the instrument’s cross-cultural applicability and long-term stability remain to be further verified.

##### Skin cancer risk perception questionnaire (Janssen et al.)

The Skin Cancer Risk Perception Questionnaire was developed by the Dutch scholar Janssen *et al*. (73) in 2011 to evaluate individuals’ subjective perceptions regarding the risk and consequences of developing skin cancer. The instrument comprises 27 items distributed across two primary dimensions: perceived likelihood and perceived severity. The structural hierarchy of the scale is detailed: the perceived likelihood dimension is stratified into absolute and comparative likelihood; absolute likelihood is further subdivided into unconditional and conditional likelihoods, both of which are bifurcated into cognitive and affective sub-dimensions. Similarly, comparative likelihood is categorized into direct and indirect comparisons, with direct comparison further delineated into cognitive and affective sub-dimensions. The perceived severity dimension comprises absolute severity, comparative severity, and specific severity; the latter is further classified into physical, social, and psychological severity. Items are rated on a 5-point Likert scale, while sun protection behaviors are scored separately. Internal consistency testing revealed significant variability across dimensions, with Cronbach’s *α* coefficients ranging widely from 0.13 to 0.90. Specifically, the unconditional likelihood and psychological severity dimensions demonstrated robust internal consistency, indicating stable and reliable measurement properties. Conversely, dimensions such as conditional cognitive likelihood yielded low α coefficients, suggesting insufficient internal consistency and weak inter-item correlations, which points to a need for optimized item design or a re-evaluation of the dimensional structure. Furthermore, given the suboptimal consistency of certain subscales and the fact that the validation sample was predominantly composed of highly educated Dutch individuals, further research is required to verify the instrument’s cross-cultural applicability, the stability of specific constructs, and its universality across different health behaviors.

##### Risk perception scale for liver cancer in patients with cirrhosis

The Risk Perception Scale for Liver Cancer in Patients with Cirrhosis was developed by Peng *et al*. (74) in 2024 to specifically evaluate the subjective perception of liver cancer risk among patients with cirrhosis. Constructed based on the Health Belief Model (47), the instrument comprises 33 items distributed across three dimensions: disease knowledge cognition, self-health management, and perceived barriers. Assessment utilizes a 5-point Likert scale; specifically, the “perceived barriers” dimension employs reverse scoring ranging from “no confidence” to “very confident,” while the remaining dimensions are scored from “strongly disagree” to “strongly agree” (1 to 5 points). The total score ranges from 33 to 165, with higher scores indicating a heightened level of risk perception regarding liver cancer. Reliability analysis demonstrated robust internal consistency, with Cronbach’s *α* coefficients ranging from 0.859 to 0.952 for the individual dimensions and 0.956 for the total scale. As the first systematic assessment tool in China targeting liver cancer risk perception in cirrhotic patients, it demonstrates high clinical applicability and cultural adaptability, with a scientifically rigorous development process and psychometric properties that meet standard criteria. However, the significant disparity in the number of items across dimensions may compromise the structural balance and interpretability of the scale. Consequently, future research should prioritize cross-regional validation in broader populations and further optimize the dimensional structure to enhance its practical utility.

#### Assessment tools for diabetes-related risk perception

##### Risk perception survey for developing diabetes (RPS-DD)

The Risk Perception Survey for Developing Diabetes (RPS-DD) was developed by Walker *et al*. (75) in 2003 to evaluate individuals’ subjective perception regarding their risk of developing diabetes. Constructed upon the theoretical frameworks of the Risk Perception Model (76) and Optimism Bias Theory (77), the final instrument comprises 53 items organized into four subscales: comparative disease risk, comparative environmental risk, optimistic bias, and personal control. The scale primarily utilizes a 4-point Likert scoring system, ranging from 1 (“almost no risk”) to 4 (“high risk”). Scores for each subscale are calculated independently, with higher scores indicating a heightened level of risk perception within that specific dimension. The Cronbach’s *α* coefficients for the subscales range from 0.64 to 0.86. However, the internal consistency reliability for the “optimistic bias” and “personal control” subscales was found to be slightly suboptimal. To date, the scale has been validated in various populations, including those in China (78) and Italy (79).

##### Risk perception survey–diabetes mellitus (RPS-DM)

The Risk Perception Survey–Diabetes Mellitus (RPS-DM) was developed by Walker *et al*. (80) in 2007 to evaluate the comparative subjective perception of diabetes complications and related health risks among diabetic patients. Constructed upon the theoretical frameworks of the Risk Perception Model (76) and Optimism Bias Theory (77), the instrument comprises 31 items distributed across six dimensions: personal control, optimistic bias, personal disease risk, environmental risk, worry about complications, and risk knowledge. The scale does not utilize a composite total score; rather, each subscale is analyzed independently, with higher scores indicating elevated levels of risk perception or knowledge within that specific dimension. Psychometric analysis revealed Cronbach’s *α* coefficients ranging from 0.64 to 0.88 for the individual dimensions, with a total scale coefficient of 0.85. The RPS-DM has been validated in various populations, including those in Iran (81), South Korea (82), China (83), and Singapore (84).

##### Perception of risk factors for type 2 diabetes mellitus scale (PRF-T2DM)

The Perception of Risk Factors for Type 2 Diabetes Mellitus Scale (PRF-T2DM) was developed by the American scholar Valmi D. Sousa *et al*. (85) in 2009 to assess individuals’ subjective perception of risk factors associated with type 2 diabetes. Grounded in the Health Belief Model (47), the scale consists of 12 items divided into two dimensions: personal and behavioral risk factors, and environmental risk factors. Items are rated on a 4-point Likert scale ranging from 0 (“do not know”) to 3 (“increases risk”), yielding a total score range of 0 to 36, where higher scores indicate a stronger perception of risk factors for type 2 diabetes. The total Cronbach’s *α* coefficient for the scale is 0.81, while the coefficients for the two dimensions are 0.74 and 0.80, respectively.

##### Perception of risk of chronic kidney disease scale (PRCKDS)

The Perception of Risk of Chronic Kidney Disease Scale (PRCKDS) was developed by the Chinese scholar Cao *et al*. (25) in 2022 to evaluate the subjective perception of concurrent chronic kidney disease (CKD) risk among patients with type 2 diabetes. Constructed using the Health Belief Model (47) as its theoretical framework, the final instrument comprises 36 items distributed across five core dimensions: susceptibility, severity, benefits, barriers, and cues to action. The scale utilizes a 4-point Likert scoring system ranging from 1 (“strongly disagree”) to 4 (“strongly agree”). Dimension scores are standardized to a 10-point scale, while the total score ranges from 36 to 144, with higher scores reflecting a higher level of CKD risk perception. Reliability testing demonstrated Cronbach’s *α* coefficients between 0.831 and 0.906 for the dimensions, and 0.875 for the total scale. By systematically assessing the multidimensional structure of risk perception across cognitive, attitudinal, and behavioral motivation levels, this scale provides a scientific basis for formulating precise and stratified health intervention strategies. Future research should prioritize multi-center, large-sample empirical studies to further facilitate the revision and application of this instrument across diverse populations and cultural contexts.

#### Assessment tools for risk perception in other diseases

##### Risk perception questionnaire (RPQ)

The Risk Perception Questionnaire (RPQ) was developed by Contreras-Yáñez *et al*. (86) in 2019 to evaluate the subjective perception of disease-related risks among Spanish-speaking patients with rheumatoid arthritis. The questionnaire comprises 27 items distributed across five dimensions: likelihood of articular and extra-articular manifestations, perception of complications/comorbidities and disease severity, likelihood of negative socioeconomic consequences, perception of personal responsibility and prevention, and perception of disease control. Scoring utilizes a Visual Analogue Scale (VAS) ranging from 0 to 100 mm per item; the total score is calculated as the mean of all items (theoretical range: 0–100), with higher scores indicating elevated levels of risk perception. Reliability testing demonstrated Cronbach’s *α* coefficients ranging from 0.81 to 0.93 for individual dimensions, with a total scale coefficient of 0.90. While the scale demonstrates good generalizability, its cross-cultural applicability and long-term stability require further verification.

##### Recurrence risk perception scale for adult patients with inflammatory bowel disease

The Recurrence Risk Perception Scale for Adult Patients with Inflammatory Bowel Disease was developed by Liu *et al*. (87) in 2024 to assess the subjective perception of disease recurrence risk in this population. Grounded in the Health Belief Model (47), the scale comprises 29 items across six dimensions: perceived susceptibility, perceived benefits of behavioral change, perceived severity, cues to health behavior, perceived barriers to behavioral change, and warning symptoms. All items use a 5-point Likert scale ranging from 1 (“strongly disagree”) to 5 (“strongly agree”). Notably, the “perceived barriers” dimension is reverse-scored—where lower scores indicate a stronger perception of recurrence risk—while other dimensions are positively scored. Cronbach’s *α* coefficients ranged from 0.834 to 0.882 for the dimensions, with an overall coefficient of 0.908. This instrument deeply reflects patients’ subjective cognitive structures and motivations for behavioral change, facilitating the identification of perceived benefits, barriers, and cues in the adoption of health behaviors, thereby providing an effective tool for targeted health education and personalized behavioral interventions.

##### Risk perception questionnaire for acute asthma exacerbation in adults

The Risk Perception Questionnaire for Acute Asthma Exacerbation in Adults was developed by Sun *et al*. (88) in 2024 to evaluate the subjective perception of acute exacerbation risk among adult asthma patients. Constructed based on the Health Belief Model (47) and Risk Perception Theory (49, 50), the questionnaire consists of 25 items organized into five dimensions: perceived susceptibility, perceived severity, perceived benefits, perceived barriers, and self-efficacy. Items are rated on a 5-point Likert scale, with options ranging from “very consistent” to “very inconsistent” assigned values of 5 to 1, respectively; higher total scores indicate a higher level of risk perception regarding acute asthma attacks. Cronbach’s *α* coefficients for the dimensions ranged from 0.773 to 0.948, with an overall coefficient of 0.918. However, as the development process was limited to patients from a single hospital, the scale’s cross-population applicability and long-term stability require further verification through multi-center, large-sample studies. Table 2 provides an overview of the key features of these disease-specific instruments.

**Table 2:** Summary of disease-specific risk perception assessment instruments.

Disease Category	Instrument name	Author (Year) (Ref)	Dimensions/Constructs	Psychometric properties (Cronbach’s *α*)
Cardiovascular	PRCHD	Becker and Levine (1987) (51)	Absolute risk, relative risk, worry/anxiety.	Total: 0.80
	PRHDS	Ammouri & Neuberger (2008) (54)	Dread risk, general risk, unknown risk.	Total: 0.80; Dims: 0.68–0.80
	CRIP	Barnhart et al. (2009) (61)	Worry, self-efficacy, susceptibility, health status.	Total: 0.76
	ABCD Risk	Woringer et al. (2017) (23)	Knowledge, perceived risk, benefits/intention, diet intention.	Dims: 0.56–0.85
Stroke	Stroke recurrence scale	Lin et al. (2021) (65)	Recurrence probability, severity, risk factors (disease/behavioral).	Section 2: 0.85
	Ischemic stroke scale	Han et al. (2022) (12)	Warning symptoms, risk factors, health risks, psychosocial risks.	Total: 0.91; Dims: 0.83–0.95
	High-risk stroke Q.	Ren et al. (2023) (67)	Susceptibility, symptoms, risk factors, control, severity.	Total: 0.88; Dims: 0.80–0.91
Oncology	Breast *Ca.* recurrence	Li et al. (2023) (68)	Likelihood, symptoms, risk factors, severity.	Total: 0.94; Dims: 0.82–0.90
	Lymphedema risk	Ma et al. (2023) (69)	Cognitive judgment, emotional response, perceived severity.	Total: 0.75; Dims: 0.87–0.89
	Skin cancer Q.	Morales-Sánchez (2014) (72)	Affect, behavior, severity, susceptibility.	Total: 0.82; Dims: 0.65–0.88
	Liver cancer (Cirrhosis)	Peng et al. (2024) (74)	Knowledge cognition, Self-management, perceived barriers.	Total: 0.96; Dims: 0.86–0.95
Diabetes	RPS-DD	Walker et al. (2003) (75)	Comparative risk, environmental risk, optimistic bias, control.	Dims: 0.64–0.86
	PRCKDS (Kidney)	Cao et al. (2022) (25)	Susceptibility, severity, benefits, barriers, cues to action.	Total: 0.88; Dims: 0.83–0.91

Ca. = Cancer; Q. = Questionnaire; Dims = Dimensions.

## Discussion

### Summary of main findings

This narrative review identified 25 instruments for assessing disease risk perception in patients with chronic diseases. These include five generic tools and 20 disease-specific tools covering cardiovascular disease, stroke, cancer, diabetes, and other conditions. These instruments vary in their theoretical foundations, dimensional structures and psychometric properties. The findings confirm a shift from unidimensional to multidimensional constructs, and from generic to disease-specific assessments, over the past several years.

### Content comparison of assessment tools

A comparative overview of the basic characteristics of risk perception assessment tools for chronic diseases is presented in Table 3. Existing instruments exhibit distinct variations regarding target populations and research contexts.

**Table 3:** Comparative characteristics of risk perception assessment tools for chronic diseases.

No.	Instrument name	Country/First author	Year	Dimensions	Theoretical framework	Cronbach’s *α*	Validation/Adaptation
1	NCD-PR5-21	Myanmar / Mya	2021	Susceptibility, benefits, barriers, self-efficacy, intention to change behavior	HBM	0.683–0.854	None
2	PRCHD	USA / Becker and Levine	1987	Absolute risk, relative risk, worry frequency	HBM	0.803	South Korea
3	PRCKDS	China / Cao	2022	Susceptibility, severity, benefits, barriers, cues to action	HBM	0.875	None
4	RPS-LC-PC^†^	China / Peng	2024	Disease knowledge, self-health management, perceived barriers	HBM	0.956	None
5	RRPS-IBD^†^	China / Liu	2024	Susceptibility, benefits, severity, cues to action, barriers, warning symptoms	HBM	0.908	None
6	PRF-T2DM	USA / Sousa	2009	Personal/behavioral risk factors, environmental risk factors	HBM	0.81	None
7	SCRPQ^†^	Mexico / Morales-Sánchez	2014	Affect, behavior, severity, susceptibility, probability	Risk Perception Theory	0.824	None
8	RRPS-IS^†^	China / Han	2022	Warning symptoms, risk factors, health risks, psychosocial risks	HBM & Risk Perception Theory	0.905	None
9	RPQ-AAE^†^	China / Sun	2024	Susceptibility, severity, benefits, barriers, self-efficacy	HBM & Risk Perception Theory	0.918	None
10	SPRR-PAS^†^	China / Lin	2021	Pt 1: Recurrence probability Pt 2: Severity, disease risk factors, behavioral risk factors	HBM & RPA	Pt 2: 0.850	None
11	BCRR-PAQ^†^	China / Li	2023	Likelihood, warning symptoms, disease risk factors, behavioral risk factors, psychological risk factors, severity	HBM & RPA	0.94	None
12	RPS-DD	USA / Walker	2003	Comparative disease risk, comparative environmental risk, optimistic bias, personal control	Risk Perception Theory & Optimism Bias	0.64–0.86	China, Italy
13	RPS-DM	USA / Walker	2007	Personal control, optimistic bias, personal disease risk, environmental risk, worry, knowledge	Risk Perception Theory & Optimism Bias	0.85	Iran, South Korea, China, Singapore
14	RPQ-Stroke^†^	China / Ren	2023	Susceptibility, symptoms, risk factors, risk control, severity	HBM & Psychometric Paradigm	0.878	None
15	PRHDS	Jordan, USA / Ammouri and Neuberger	2008	Dread risk, general risk, unknown risk	Psychometric Paradigm	0.8	Jordan, USA, China, Italy, Nigeria
16	RPS-DA-OP^†^	China / Wang	2023	Affective experience, possible causes, severe consequences, behavioral control	CEST, Affect Heuristic & Risk Perception Theory	0.973	None
17	LRPQ-BC^†^	China / Ma	2023	Cognitive judgment, emotional response, perceived severity	Dual-Process Model	0.745	None
18	CRIP	USA / Barnhart	2009	Worry, self-efficacy, susceptibility/vulnerability, health status	/	0.76	None
19	ABCD Risk	UK / Woringer	2017	Knowledge, risk perception, benefits & intention, healthy eating intention	HBM & TTM	Risk: 0.85Benefits: 0.82Diet: 0.56	Netherlands, China, Malaysia, Indonesia, UK, Jordan, Egypt
20	CPRP	China / Fang	2015	Economic risk, physical diagnosis/treatment risk, psychosocial risk	/	0.884	None
21	TRIRISK	USA / Ferrer	2016	Deliberative, affective, experiential	/	0.93	China, UK
22	RPQ (RA)	Mexico / Contreras-Yáñez	2019	Likelihood, severity, socioeconomic consequences, personal responsibility & prevention, disease control	/	0.9	None
23	RPCD	USA / Hamilton	2012	Personal risk, other’s risk, prevalence estimation, mortality estimation	/	0.62–0.82	None
24	SCRPQ^†^	Netherlands / Janssen	2011	Perceived likelihood, perceived severity	/	0.13–0.90	None
25	VAS	Iran / Hajian-Tilaki	2021	Single item	/	N/A	N/A

^†^ Indicates scales where an English acronym was not explicitly provided in the original text; a descriptive abbreviation has been assigned for tabular clarity. HBM: Health Belief Model; RPA: Risk Perception Attitude Framework; CEST: Cognitive-Experiential Self-Theory; TTM: Transtheoretical Model; N/A: Not Applicable; /: Not specified or no specific theoretical model listed.

Regarding target populations, the CPRP (9), TRIRISK (10), NCD-PR5-21 (16), RPCD (11), and the Risk Perception Scale of Disease Aggravation for Older Patients (48) are generally applicable to chronic disease contexts. However, specific distinctions exist: TRIRISK (10) is primarily designed to assess the risk perception of developing chronic diseases among healthy populations; NCD-PR5-21 (16) focuses on evaluating common risk behaviors associated with cardiovascular diseases, diabetes, cancer, and chronic respiratory diseases; RPCD (11) targets young and middle-aged women; and while the Risk Perception Scale of Disease Aggravation for Older Patients (48) was developed specifically for older adults, it is applicable across a spectrum of chronic conditions. Furthermore, the PRCHD (51) is specifically utilized to assess risk perception among unaffected siblings of patients with premature coronary heart disease, whereas the development and validation of the CRIP (61) have been confined to urban minority populations in the United States.

**Figure 3:**
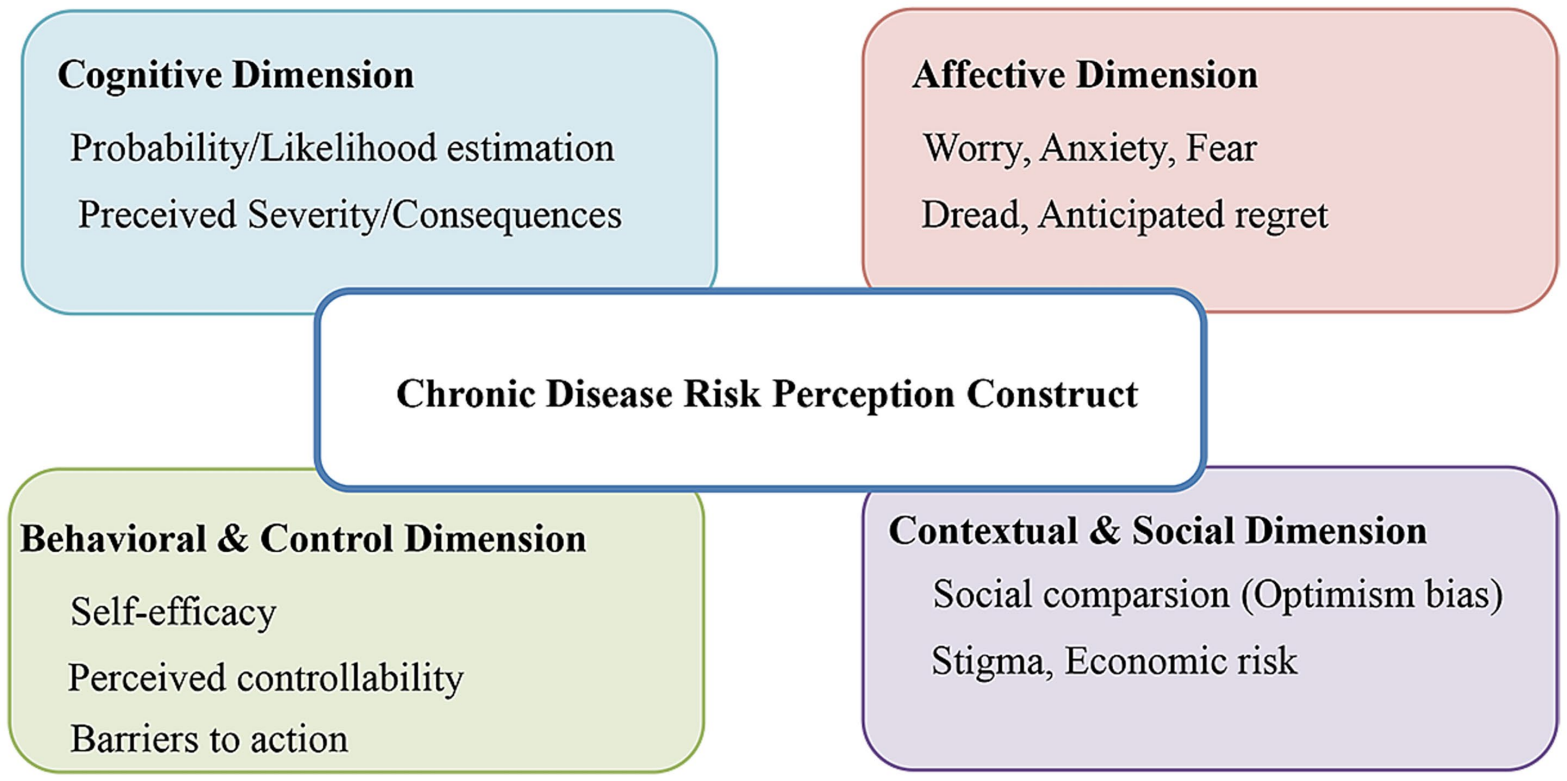
The multidimensional construct of risk perception. The multidimensional construct of chronic disease risk perception derived from the content analysis of reviewed instruments. The diagram highlights that contemporary risk perception is not a unitary concept but an integration of cognitive judgments, affective responses, behavioral beliefs, and contextual factor.

In terms of assessment content, the NCD-PR5-21 (16), RPCD (11), PRCHD (51), Stroke Patient Recurrence Risk Perception Assessment Scale (65), Skin Cancer Risk Perception Questionnaire (Janssen et al.) (73), VAS (71), RPS-DD (75), and RPQ (86) primarily focus on the individual’s cognitive estimation of disease occurrence probability (Table 4). Conversely, the Lymphedema Risk Perception Questionnaire for Female Breast Cancer Patients (69), Skin Cancer Risk Perception Questionnaire (Morales-Sánchez et al.) (72), PRCKDS (25), Breast Cancer Recurrence Risk Perception Assessment Questionnaire (68), and Recurrence Risk Perception Scale for Adult Patients with Inflammatory Bowel Disease (87) emphasize the assessment of perceived severity regarding disease consequences. Instruments such as the Stroke Patient Recurrence Risk Perception Assessment Scale (65), Risk Perception Questionnaire for People at High Risk of Stroke (67), Breast Cancer Recurrence Risk Perception Assessment Questionnaire (68), and PRF-T2DM (85) center on the individual’s ability to identify risk factors associated with disease onset. Finally, the CPRP (9), Risk Perception Scale of Disease Aggravation for Older Patients (48), PRHDS (54), and CRIP (61) tend to explore the interplay between individual emotional responses and cognitive risk perception. Synthesizing these diverse measurement focuses, Figure 3 illustrates the comprehensive multidimensional construct of chronic disease risk perception derived from the reviewed instruments.

**Table 4:** Content domain mapping of selected risk perception instruments.

Instrument name (Ref)	Probability / Susceptibility	Severity / Consequences	Risk Factors / Symptoms	Affective / Emotional Response
Generic tools				
CPRP (9)				•
TRIRISK (10)	•			•
RPCD (11)	•			
CVD and stroke				
PRCHD (51)	•			
PRHDS (54)				•
Stroke recurrence (Lin) (65)	•	•	•	
High-risk stroke (Ren) (67)	•	•	•	
Oncology				
Breast *Ca.* recurrence (68)	•	•	•	
Lymphedema risk (69)		•		•
Skin cancer (Janssen) (73)	•	•		
Diabetes				
RPS-DD (75)	•			• (Bias)
PRCKDS (25)	•	•		
PRF-T2DM (85)			•	

• indicates the instrument explicitly includes a distinct dimension or sub-scale measuring this domain. Analysis: While most tools measure Probability (Susceptibility), there is a distinct divergence where some tools prioritize Risk Factors (e.g., stroke recurrence models) while others prioritize Affective Responses (e.g., Lymphedema, PRHDS). Future tools are encouraged to integrate all four domains for comprehensive assessment.

### Theoretical frameworks and their evolution

The Health Belief Model (HBM) (47) serves as a foundational theoretical framework for explaining individual health behaviors. Its core tenet posits that an individual’s engagement in health-promoting behaviors is modulated by their perception of health threats and their motivation to effect behavioral change. Instruments developed based on the HBM typically incorporate dimensions related to the perception of health risks. The primary objective of such scales is to evaluate an individual’s cognitive understanding of the disease and their psychological responses when confronting illness, thereby effectively assessing the perception of health threats (16, 25, 51, 74, 85, 87).

Risk Perception Theory (49, 50) places greater emphasis on the interplay between emotional reactions and rational evaluations when individuals confront health risks. This theoretical perspective posits that the perception of health risks is driven not solely by rational cognition but is also significantly influenced by affective factors. Consequently, scales grounded in Risk Perception Theory typically prioritize the assessment of emotional responses, as well as perceptions of severity and probability (72).

Optimism Bias Theory (77) approaches risk perception from the perspective of excessive optimism regarding personal health, exploring how individuals tend to underestimate their own risk of disease while overestimating the likelihood of illness in others. Scales developed upon this theory typically focus on cognitive biases in risk perception. These instruments prioritize the assessment of perceived control and risk biases—specifically, the impact of optimistic bias on health behavioral decision-making. The application of Optimism Bias Theory facilitates the elucidation of psychological biases in risk assessment, which is particularly valuable for identifying potential cognitive errors during the design of intervention strategies (75, 80).

The Dual-Process Model (70) integrates assessment at both cognitive and affective levels, emphasizing the interaction between rational judgment and emotional response when individuals face health risks. Scales developed based on this model typically encompass dimensions of both rational judgment and affective reaction, exploring how these mechanisms synergistically influence risk perception (69). In assessing health risk perception, these instruments account for both rational cognitive evaluations and irrational emotional responses. To facilitate a direct comparison, the core mechanisms, key constructs, advantages, and limitations of these predominant theoretical frameworks are summarized in Table 5.

**Table 5:** Comparative analysis of theoretical frameworks utilized in risk perception assessment.

Theoretical framework	Core mechanism/Focus	Key constructs analyzed	Advantages	Limitations	Representative tools (Ref)
Health belief model (HBM)	Rational Cognition: Focuses on the cognitive assessment of threats and the cost–benefit analysis of action.	Susceptibility, severity, benefits, barriers, cues to action, self-efficacy.	widely used; effective for assessing deliberate health behavior changes.	Overemphasizes rationality; often neglects emotional, cultural, and environmental drivers.	NCD-PR5-21 (16), PRCHD (51), PRCKDS (25)
Risk perception theory	Affective-Rational Interface: Posits that perception is driven by both statistical probability and emotional reaction (e.g., dread).	Probability, severity, dread/fear, unknown risk.	Captures the “non-rational” emotional components of risk.	May not fully account for sociocultural variations in risk interpretation.	Skin Cancer Q. (72), PRHDS (54), Asthma Q. (88)
Optimism bias theory	Cognitive Bias: Focuses on the tendency to underestimate personal risk compared to others (unrealistic optimism).	Comparative risk (Self vs. Others), Personal control, Optimistic bias.	Excellent for identifying cognitive errors and “invulnerability” myths in patients.	Less focus on affective and social determinants; narrower scope.	RPS-DD (75), RPS-DM (80)
Dual-process model	Integrated Processing: Distinguishes between and integrates “System 1” (Experiential/Affective) and “System 2” (Analytical/Deliberative).	Cognitive judgment (Rational), Emotional response (Affective).	Most holistic framework; acknowledges that emotion and reason operate in parallel.	Complexity in measurement; requires careful operationalization of constructs.	Lymphedema Risk Q. (69), TRIRISK (10)

**Figure 4:**
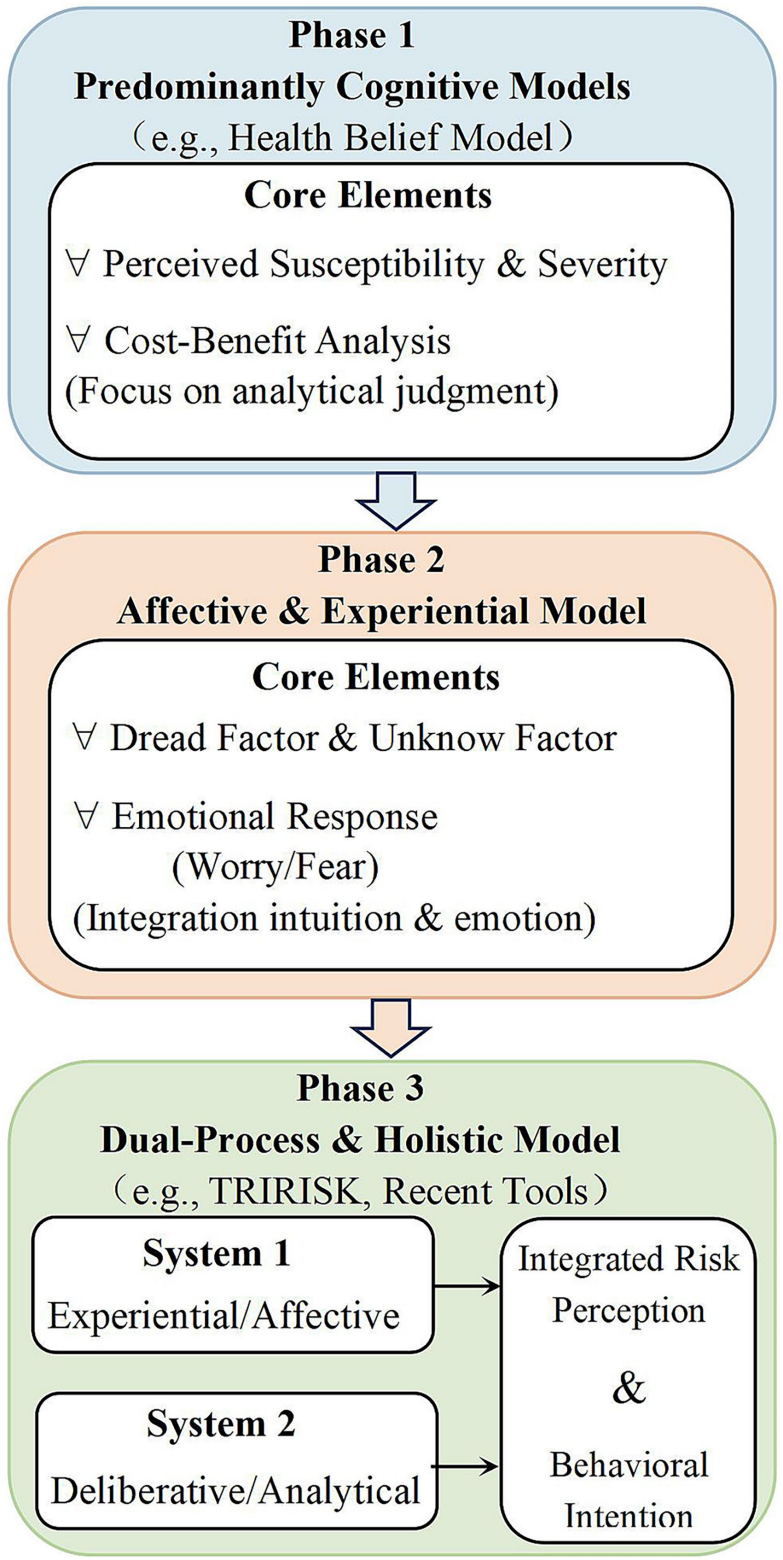
Conceptual evolution of theoretical frameworks. The conceptual evolution of theoretical frameworks underpinning risk perception assessment. The figure illustrates the paradigm shift from predominantly cognitive, rational models (e.g., HBM) toward integrated dual-process frameworks that incorporate both analytical reasoning and affective/experiential components.

Furthermore, the conceptual evolution of these theoretical frameworks highlights a shift from traditional cognitive models, which focus primarily on rational and logical processes, to more comprehensive dual-process systems that incorporate cognitive and emotional components. This shift reflects a broader understanding of how individuals process risk information, acknowledging the importance of rational analysis and emotional responses in shaping risk perceptions. This progression is depicted in Figure 4.

In summary, scales developed from these diverse models exhibit significant differences in their core theoretical underpinnings and assessment dimensions. The Health Belief Model (47) focuses on the cognition of disease threats and the motivation for behavioral change, primarily centering on dimensions such as perceived susceptibility, severity, and willingness to change behavior. In contrast, Risk Perception Theory (49, 50) emphasizes the influence of affective factors, directing attention toward emotional responses and probability perception. Optimism Bias Theory (77) prioritizes the analysis of cognitive biases—particularly the effect of optimistic bias on decision-making. Meanwhile, the Dual-Process Model (70) synthesizes cognitive and affective responses to provide a more holistic framework. Although these theoretical models possess distinct characteristics, their practical applications are often complementary. In research concerning health interventions and behavioral change, employing scales that integrate considerations from these various theoretical models allows for a more comprehensive assessment of individual risk perception, thereby providing a robust evidence base for formulating more personalized and effective health promotion strategies.

### Practical applications in clinical and research settings

The reviewed risk perception assessment tools have a number of practical applications in clinical practice and research.

In clinical settings, these instruments can be used to identify patients with inaccurate risk perceptions, whether that be overestimation, which can lead to anxiety, or underestimation, which can result in poor adherence. The CPRP (9) and TRIRISK (10) can be used to screen for maladaptive risk beliefs during initial patient assessments. In cardiovascular care, the PRCHD (51) or PRHDS (54) can help clinicians to tailor counselling for patients who perceive themselves as being at low risk of heart disease. For stroke survivors, the Recurrence Risk Perception Scale (65) or the Ischemic Stroke Recurrence Scale (12) can inform secondary prevention education by identifying knowledge gaps. In oncology, the Breast Cancer Recurrence Risk Perception Assessment Questionnaire (68) and the Lymphedema Risk Perception Questionnaire (69) for Female Breast Cancer Patients enable clinicians to address rational concerns and emotional distress, facilitating shared decision-making regarding follow-up and lifestyle modifications. The ABCD Risk Questionnaire (23) and the NCD-PR5-21 (16) are suitable for use in primary care settings to assess population-level risk awareness and evaluate the impact of health promotion campaigns. When selecting a tool for a study, researchers should consider the target disease, the theoretical framework underlying the instrument and whether cross-cultural validation is available (see Table 3). For multinational studies, tools that have already been adapted for use in multiple languages and settings are preferable.

Practical considerations for use include: (a) checking whether the tool has been validated in the target population;(b) assessing the reading level and item complexity, especially for patients who have had a stroke or are older adults;(c) considering the time taken to administer the tool — brief scales are suitable for busy clinical workflows, while more comprehensive tools may be reserved for research or specialist clinics; and (d) using the subscale scores of the tool to design targeted interventions.

Overall, these instruments are not just academic exercises; they directly facilitate patient-centered communication, risk-tailored education and the evaluation of behavioral interventions. Integrating them into routine care pathways and clinical trials can improve the effectiveness of chronic disease management.

## Limitations and implications

**Figure 5:**
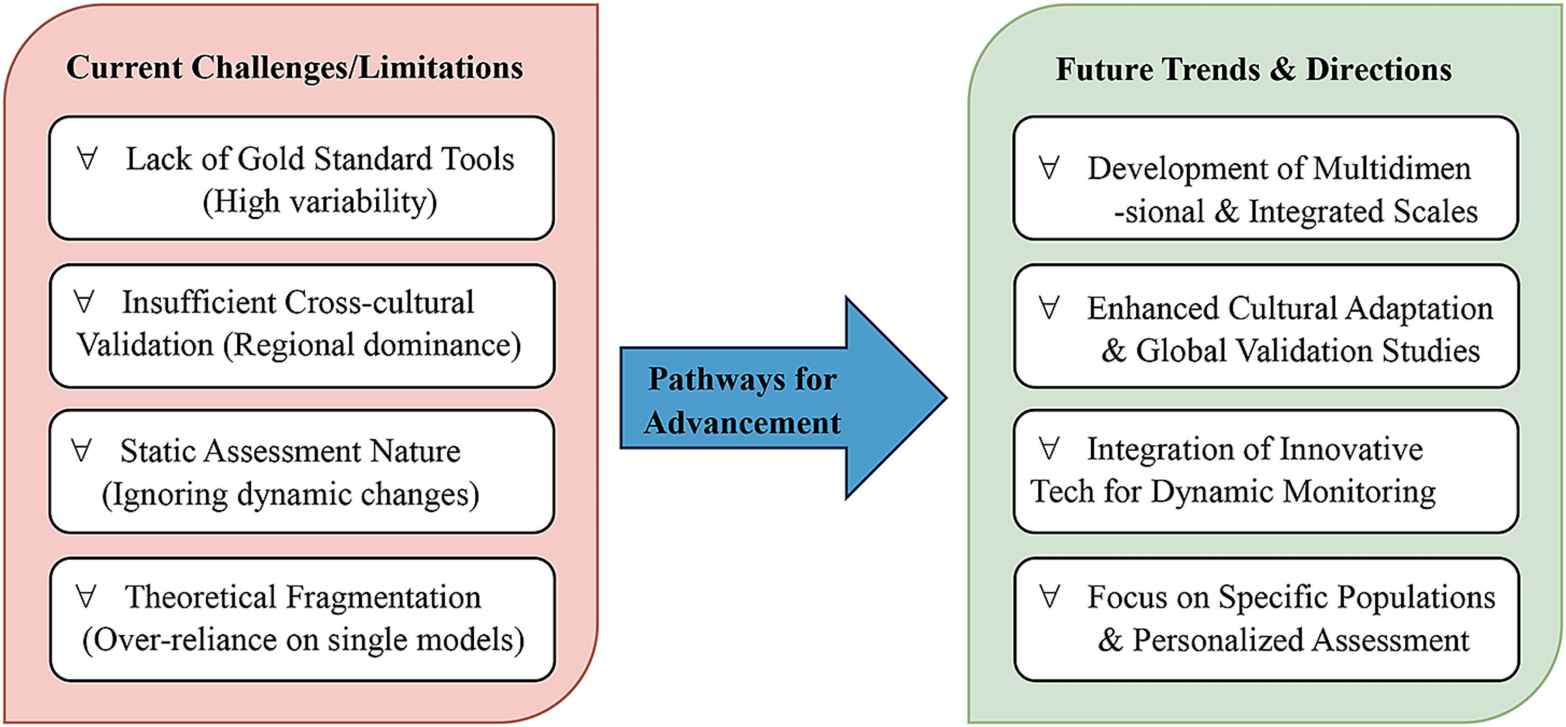
Roadmap of challenges and future directions. Summary roadmap outlining current limitations and proposed future directions in chronic disease risk perception assessment research. The figure synthesizes the critical analysis mapping the transition from existing challenges toward more dynamic, integrated, and culturally valid assessment methodologies.

Despite significant theoretical and practical advancements in research on chronic disease risk perception, several limitations persist, offering critical insights for future scholarship. Figure 5 provides a visual roadmap summarizing these current challenges and outlining pathways for future advancement, which we now discuss in detail.

First, the inadequate consideration of individual differences remains a predominant challenge. While many instruments can effectively assess risk perception in chronic disease patients, the majority fail to sufficiently account for the potential influence of cultural backgrounds, socioeconomic status, and psychological states during their design. By relying heavily on standardized questionnaires and rigid theoretical frameworks, these tools often overlook variations in risk perception across diverse sociocultural groups and geographic contexts. Consequently, extensive cross-cultural and cross-population validation is imperative to mitigate potential measurement biases. As illustrated in the left panel of Figure 5, this limitation calls for future instruments that embed cultural adaptability and personalized assessment modules.

Second, the interplay between affective factors and rational judgment remains under-explored. Although some instruments have begun to integrate these distinct elements (12, 48, 67, 68, 75, 80, 88, 89), the majority of chronic disease risk perception scales continue to prioritize unidimensional assessment, thereby neglecting the complex interaction between affective responses and cognitive evaluations. Given that affective factors often directly modulate health behaviors yet remain insufficiently examined in most studies, future research must prioritize the comprehensive assessment of both emotion and rational cognition to achieve a more holistic understanding of individual risk perception.

Third, the predictive validity regarding behavioral change represents a significant deficiency in current instruments. While many scales effectively gauge the level of risk perception, their ability to predict actual behavioral modification is often weak. Even when individuals accurately perceive their health risks, the translation of this perception into action is contingent upon multifarious factors, including self-efficacy, social support, and environmental constraints. Therefore, future instrument development should place greater emphasis on establishing strong associations with actual health behaviors, particularly regarding practical applications in chronic disease management and prevention. As can be seen in the right-hand panel of Figure 5, this requires the integration of behavioral outcome measures during scale validation, as well as the use of longitudinal designs to test predictive validity.

In summary, Figure 5 outlines these three major challenges and suggests the following research priorities: (1) cross-cultural and cross-population validation; (2) the integration of affective and rational dimensions; and (3) the enhancement of predictive validity for behavior change. Addressing these issues will improve the design and effectiveness of risk perception instruments, ultimately supporting personalized chronic disease management and intervention strategies.

## Conclusion

Research on risk perception in chronic diseases provides a critical perspective for understanding how individuals appraise their personal health threats. An analysis of existing instruments reveals that diverse theoretical frameworks offer multidimensional viewpoints for assessing risk perception among patients with chronic conditions. Frameworks such as the Health Belief Model, Risk Perception Theory, Optimism Bias Theory, and the Dual-Process Model each possess distinct characteristics and have been extensively applied in the development of risk perception scales. However, while these instruments offer profound theoretical insights, limitations persist in their practical application, particularly regarding the consideration of individual differences, the complex interplay between affective responses and rational judgments, and predictive validity for behavioral change. Future research must address these deficiencies to further refine the design and utility of these instruments. Concurrently, cross-cultural and cross-population studies should be prioritized to ensure the validity and operability of these scales on a global scale.
