# Supplementary material for: Assessment tools for disease risk perception in chronic patients: theoretical frameworks, psychometric properties, and clinical applications
Source: Front Public Health. 2026 May 8;14:1821777. doi: 10.3389/fpubh.2026.1821777 (PMC13194426; doi:10.3389/fpubh.2026.1821777)
Supplement: Supplementary file 1 [file Supplementary_File_1.PDF]

Disease Category	Instrument Name	PROM development	Content validity
Generic Disease Risk Perception Assessment Tools	CPRP	A	A
	TRIRISK	NA	D
	NCD-PR5-21	A	A
	RPCD	NA	D
	Risk Perception Scale of Disease Aggravation	G	G
Cardiovascular	PRCHD	NA	D
	PRHDS	A	G
	CRIP	NA	D
	ABCD Risk Q	A	A
Stroke	Stroke Recurrence Scale	A	G
	Ischemic Stroke Scale	G	G
	High-Risk Stroke Q.	G	G
Oncology	Breast Ca. Recurrence	G	G
	Lymphedema Risk	G	G
	VAS	NA	D
	Skin Cancer Q.*(Morales-Sánchez et al.)*	A	D
	Skin Cancer Q.(*Janssen et al*.)	NA	NA
	Liver Cancer (Cirrhosis)	G	G
Diabetes	RPS-DD	NA	D
	RPS-DM	NA	D
	PRF-T2DMA	NA	D

	PRCKDS (Kidney)	A	A
Other Diseases	RPQ	G	G
	Inflammatory Bowel Disease Recurrence	G	G
	Acute Asthma Exacerbation Q	G	G

Ca. = Cancer; Q. = Questionnaire; Dims = Dimensions;G = Vary good; A = Adequate; D = D

Structural validity	Internal consistency	Cross-cultural validity	Reliability	Measurement error
A	G	NA	G	I
G	G	NA	NA	NA
G	G	NA	NA	NA
A	NA	G	NA	NA
G	G	NA	G	I
NA	G	NA	NA	NA
A	G	NA	G	I
NA	G	NA	NA	NA
A	A	NA	NA	NA
A	G	NA	A	I
G	G	NA	G	I
G	G	NA	G	I
G	G	NA	G	I
G	G	NA	NA	NA
NA	NA	NA	NA	NA
A	G	NA	NA	NA
NA	A	NA	A	NA
G	G	NA	I	I
NA	A	NA	NA	NA
NA	A	NA	A	NA
G	G	NA	NA	A

G	G	NA	NA	NA
G	G	NA	G	I
G	G	NA	G	I
G	G	NA	G	I

oubtful; I = inadequate.

Criterion validity	Hypotheses testing for construct validity	Responsiveness
NA	A	NA
NA	A	NA
NA	A	NA
NA	I	NA
NA	A	NA
NA	A	NA
NA	G	NA
NA	A	NA
NA	D	NA
NA	D	NA
G	A	NA
NA	A	NA
NA	A	NA
NA	A	NA
I	A	NA
NA	D	NA
NA	G	NA
NA	A	NA
NA	A	NA
NA	D	NA
NA	G	NA

NA	A	NA
A	A	NA
G	A	NA
A	A	NA
