# Supplementary material for: Assessment tools for disease risk perception in chronic patients: theoretical frameworks, psychometric properties, and clinical applications
Source: Front Public Health. 2026 May 8;14:1821777. doi: 10.3389/fpubh.2026.1821777 (PMC13194426; doi:10.3389/fpubh.2026.1821777)
Supplement: Supplementary file 2 [file Supplementary_File_2.PDF]

Wan fang：

#1:主题：（风险感知+感知风险+风险认知+风险评估+风险判断）

#2:主题：（量表+问卷+评分+调查+研究工具+测评工具）

#3:#1 AND #2

China National Knowledge Infrastructure (CNKI)：

#1:SU=（风险感知+感知风险+风险认知+风险评估+风险判断）

#2:SU=（量表+问卷+评分+调查+研究工具+测评工具）

#3:#1 AND #2

China Science and Technology Journal (VIP)：

#1:M=风险感知 OR M=感知风险 OR M=风险认知 OR M=风险评估 OR M=风险判断

#2:M=量表 OR M=问卷 OR M=评分 OR M=调查 OR M=研究工具 OR M=测评工具

#3:#1 AND #2

PUBMED：

#1:“risk perception”[tiab] OR “perceived risk”[tiab] OR “risk awareness”[tiab] OR “risk assessment”[tiab] OR “risk factors”[tiab] OR “risk score”[tiab] OR “perceived worry”[tiab] OR “fear'[tiab] OR “percept*”[tiab] OR “perceiv*”[tiab] OR “risk*”[tiab]

#2:“Surveys and Questionnaires"[mesh] OR “measure”[tiab] OR “scale”[tiab] OR “metric”'[tiab] OR “index”[tiab] OR “instrument”[tiab] OR “tool”[tiab] OR “survey”[tiab] OR “questionnaire”[tiab] OR “score”[tiab] OR “subscale”[tiab] OR “domain”[tiab] OR “model”[tiab]

#3:#1 AND #2

Cochrane Library：

#1:('risk perception' OR 'perceived risk' OR 'risk awareness' OR 'risk assessment' OR 'risk factors' OR 'cancer, lung' OR 'risk score' OR 'perceived worry' OR 'fear'):ti

#2: ('Surveys and Questionnaires' OR 'measure' OR 'scale' OR 'metric' OR 'index' OR 'instrument' OR 'tool' OR 'survey' OR 'questionnaire' OR 'score' OR 'subscale' OR 'domain' OR model'):ti

#3:#1 AND #2

Web of Science（WOS）：

#1:TS=“risk perception' OR “perceived risk' OR “risk awareness' OR “risk assessment' OR “risk factors' OR “risk score' OR “perceived worry' OR “fear' OR “percept*' OR “perceiv*' OR “risk*'

#2:TS=“Surveys and Questionnaires" OR “measure' OR “scale' OR “metric' OR “index' OR “instrument' OR “tool' OR “survey' OR “questionnaire' OR “score' OR “subscale'[tiab] OR “domain' OR “model'

#3:#1 AND #2

Embase：

#1:('risk perception':ti,ab,kw OR 'perceived risk':ti,ab,kw OR 'risk awareness':ti,ab,kw OR 'risk assessment':ti,ab,kw OR 'risk factors':ti,ab,kw OR 'risk score':ti,ab,kw OR 'perceived worry':ti,ab,kw OR 'fear':ti,ab,kw OR 'percept*':ti,ab,kw OR 'perceiv*':ti,ab,kw OR 'risk*':ti,ab,kw)

#2:('surveys' AND 'questionnaire'/exp OR 'measure':ti,ab,kw OR 'scale':ti,ab,kw OR 'metric':ti,ab,kw OR 'index':ti,ab,kw OR 'instrument':ti,ab,kw OR 'tool':ti,ab,kw OR 'survey':ti,ab,kw OR 'questionnaire':ti,ab,kw OR 'score':ti,ab,kw OR 'subscale':ti,ab,kw OR 'domain':ti,ab,kw OR 'model':ti,ab,kw)

#3:#1 AND #2

Cumulative Index to Nursing Allied Health Literature (CINAHL)：

#1:TI(“risk perception” OR “perceived risk” OR “risk awareness”OR “risk assessment” OR “risk factors” OR “risk score” OR “perceived worry” OR “fear” OR “percept*” OR “perceiv*”OR “risk*”)

#2:SU(“Surveys and Questionnaires") OR TI(“measure” OR “scale” OR “metric” OR “index” OR “instrument” OR “tool” OR “survey” OR “questionnaire” OR “score” OR “subscale” OR “domain” OR “model”)

#3:#1 AND #2
